# A *Chlorella pyrenoids* Hexapeptide VPIIMH Alleviates Lipid Accumulation and Oxidative Stress in *Caenorhabditis elegans*: Insight from In Vitro, In Vivo, and Network Parmacology Analyses

**DOI:** 10.3390/foods15111965

**Published:** 2026-06-02

**Authors:** Luan Lin, Lan Luo, Haihao Guo, Yanyan Wang, Ziqing Yu, Hongya Sun, Jingyue Yao, Peng Liang, Baobei Wang

**Affiliations:** 1 Key Laboratory of Coastal Marine Biotechnology for Universities in Fujian Province, College of Oceanology and Food Science, Quanzhou Normal University, Quanzhou 362000, China; luan9008@qztc.edu.cn (L.L.); lan040923@163.com (L.L.); guo007_1101@163.com (H.G.); 231004070@stumail.qztc.edu.cn (Y.W.); shennanmu1@163.com (Z.Y.); 18263168005@163.com (H.S.); 15141098210@163.com (J.Y.); liangpeng137@sina.com (P.L.); 2 Fujian Province Key Laboratory for the Development of Bioactive Material from Marine Algae, College of Oceanology and Food Science, Quanzhou Normal University, Quanzhou 362000, China; 3 College of Food Science, Fujian Agriculture and Forestry University, Fuzhou 350002, China

**Keywords:** *Chlorella pyrenoidosa*, *Caenorhabditis elegans*, lipid-lowing, anti-obesity, network pharmacology, hexapeptide

## Abstract

Plant-derived bioactive peptides have garnered widespread interest for their functions in managing obesity and associated metabolic disorders. This study investigated the lipid-lowering activity and underlying mechanisms of VPIIMH, a hexapeptide derived from *Chlorella pyrenoids*, using in vitro enzymatic assays, *Caenorhabditis elegans* models, and network pharmacology. In vitro, VPIIMH acted as a reversible non-competitive inhibitor of pancreatic lipase, achieving an inhibition rate of 43.17 ± 1.47% at 8.0 mg/mL. Molecular docking revealed that this inhibition likely occurs through ionic bonds between VPIIMH and PL (1LPB) at Arg256. In a high-fat *C. elegans* model, treatment with 0.5 mg/mL VPIIMH significantly reduced fat accumulation by 37.2% and triglyceride levels by 26.9%. Furthermore, VPIIMH extended the lifespan of *C. elegans* under oxidant stress by 40.3% and under heat stress by 17.5%. Network pharmacology predicted that VPIIMH targets nine core proteins, which were classified into three synergistic modules: the SIRT1-PPAR for core regulation, the RAS for systemic coordination, and the inflammatory target (CCR5, MMP9, EGFR) for microenvironment support. This study elucidates the multi-target and multi-pathway mechanism of VPIIMH, suggesting its potential application in combating obesity and related lipid metabolism disorders. These findings provide a scientific basis for the development of VPIIMH as a functional food ingredient targeting metabolic health.

## 1. Introduction

Approximately 40% of adults worldwide are affected by obesity-related health problems. Obesity is primarily caused by an imbalance between energy intake and expenditure [1]. Long-term obesity can trigger a series of metabolic syndromes, triggering metabolic syndrome, significantly increasing the risks of diabetes, hypertension, dyslipidemia, atherosclerosis, and cancer [2]. Although lipid-lowering drugs such as orlistat, statins, and fibrates have shown great clinical efficacy, long-term use of these drugs can cause adverse reactions, including nausea, abdominal distension, and muscle pain [3]. Therefore, bioactive peptides derived from natural sources have attracted widespread attention because of their long-lasting effect and low side effects, and they are increasingly recognized as promising candidates for the development of functional foods targeting metabolic disorders.

Studies have identified lipid-regulating peptides from various foods, including hydrolysates of milk proteins [4], soybeans [5], sardines [6], and oysters [7]. *Chlorella*, a novel food resource in China [8], is rich in plant protein (55–65%) and contains all 18 essential amino acids, representing an excellent source for bioactive peptides preparation. A decapeptide (LLVVYPWTQR) from *Chlorella pyrenoidosa* exhibited potent porcine pancreatic lipase (PL) inhibitory activity (47.95% at 200 μg/mL). This decapeptide (600 µg/mL) significantly reduced intracellular triglycerides (TG) accumulation (27.9% inhibition) and suppressed lipogenesis through pathways associated with non-alcoholic fatty liver disease and the AMPK signaling pathway in 3T3-L1 cells [9]. Wang et al. [10] identified a pentapeptide (VWTPI) from *Chlorella pyrenoidosa* and found it inhibited 23.25% of PL activity at 2 mg/mL.

PL is the crucial enzyme responsible for dietary TG hydrolysis in the gastrointestinal tract, digesting approximately 70% of dietary fats into free fatty acids and monoacylglycerol for absorption [11,12]. Due to its local action in the intestine, PL inhibition represents an effective strategy for reducing TG absorption and managing obesity [1]. Currently, orlistat is the only FDA-approved PL inhibitor for clinical use, but its irreversible inactivation of the enzyme and associated gastrointestinal side effects limit its long-term tolerability [11,12,13]. These limitations have prompted the search for safer, reversible PL inhibitors from natural sources. Food-derived bioactive peptides have emerged as a new class of PL inhibitors, offering advantages such as mild effects, minimal side effects, and cost-effectiveness [12].

In our previous study [14], we screened multiple novel PL-inhibitory peptides from a *C. pyrenoidosa* protein hydrolysate prepared by papain. Among them, the hexapeptide VPIIMH existed as a promising candidate for further investigation. The present study focuses on VPIIMH to systematically investigate its lipid-lowering activity and potential molecular mechanism. Specifically, we evaluated its inhibitory activity and kinetic characteristics against PL through an in vitro assay, assessed its effect on lipid metabolism using the high-fat model of *Caenorhabditis elegans* induced by glucose, and employed network pharmacology to predict the key targets and pathways involved. This study aims to provide a theoretical basis for the development of VPIIMH as a functional food ingredient.

## 2. Materials and Methods

### 2.1. Materials

The hexapeptide VPIIMH, whose sequence was derived from the *Cholrella pyrenoidosa* protein, was chemically synthesized by Sangong Biotech Co., Ltd. (Shanghai, China) with a purity of >95%; wild-type *C. elegans* N2 was obtained from Fujian Shangyuan Biotechnology Co., Ltd. (Fuzhou, China); Oil Red O staining solution was purchased from Sangong Biotech Co., Ltd. (Shanghai, China). All other reagents of analytical grade were purchased from Sinopharm Chemical Reagent Co., Ltd. (Shanghai, China).

### 2.2. PL Inhibitory Activity Determination

The PL inhibitory activity was determined according to our previously reported method [10]. Briefly, PL solution, sample solution, and Tris-HCl buffer were mixed and pre-incubated, followed by the immediate addition of the substrate palmitic acid p-nitrophenyl ester (pNPP). The absorbance was read at 405 nm after termination by heat (100 °C for 5 min). The control group replaced the sample with buffer, and the blank group replaced the enzyme with buffer. Orlistat (8 μg/mL) was used as a positive control. The PL inhibitory rate was calculated using the following equation:

$$\mathrm{PL}\;\mathrm{inhibitory}\;\mathrm{rate}\;\left(\%\right)=(1-\frac{A_{s}-A_{s0}}{A_{c}-A_{c0}})\times 100$$

where *A*_*c*_ and *A*_*c*0_ are the absorbances of the control test and its blank; *A*_s_ and *A*_s0_ are the absorbances of the sample and its blank, respectively.

### 2.3. Inhibition Kinetics of PL

The inhibition kinetics of VPIIMH against PL were investigated following our previous study [10].

For the reversible inhibition assay, the substrate concentration (pNPP) was fixed at 0.8 mmol/L, and PL concentration was varied (0, 5, 10, 15, 20 mg/mL) in the absence or presence of 1 mg/mL VPIIMH. The reaction product (pNP) was quantified using a pNP standard curve to calculate the initial reaction velocities.

For the Lineweaver–Burk double-reciprocal plots, enzymatic activities were measured at substrate concentrations of 1.0, 2.0, 4.0, and 8.0 mmol/L pNPP in the presence of VPIIMH at 0.0, 1.0, and 2.0 mg/mL. The inhibition type and kinetic parameters (*V*_max_ and *K*_m_) were calculated from the plots.

### 2.4. Molecular Docking

The human pancreatic triacylglycerol lipase (PL) (PDB code: 1LPB) was obtained from the RCSB Protein Data Bank (www.rcsb.org/). The docking simulation was carried out using Molecular Operating Environment (MOE v2018.01) software with the flexible induced fit mode [10]. The conformation with the lowest binding energy was visualized using PyMOL 3.0.0.

### 2.5. Evaluation of Lipid-Lowering Activity Through the Experiment with C. elegans

#### 2.5.1. Cultivation, Synchronization, and Drug Treatment of *C. elegans*

*C. elegans* were maintained on nematode growth medium (NGM) agar plates seeded with *E. coli* OP50 at 20 °C. Synchronized L4-stage nematodes were obtained by hypochlorite treatment of gravid adults according to Liu et al. [15]. To inhibit progeny production, 5-fluoro-2′-deoxyuridine was added to the medium at 12.5 μg/mL during the formal experiment [14].

Synchronized L4-stage nematodes were divided into six groups (Table 1). The high-fat model group was established by NGM containing 10 mM glucose. VPIIMH solutions were prepared by mixing with *E. coli* OP50 (1:9, *v*/*v*) to give 0.125, 0.25, and 0.5 mg/mL. Orlistat was mixed with OP50 (1:9, *v*/*v*) to give 6 μg/mL as a positive control. The control group received OP50 mixed with sterile water. Drugs were administered via feeding, and the drug-containing bacterial mixtures (1200 μL) were spread onto NGM plates and cultured at 20 °C for 24 h prior to use [16]. Plates were replaced daily, and nematodes were collected after 72 h for analysis.

#### 2.5.2. Oil Red O Staining

Oil Red O staining was conducted as previously described [14]. Briefly, after 72 h of treatment, *C. elegans* were collected, fixed, and stained with Oil Red O solution. Stained *C. elegans* were observed and imaged using a microscope. For quantification, the dye was extracted with ethanol, and the absorbance was read at 510 nm.

#### 2.5.3. TG Content Measurement

Synchronized L4-stage nematodes were cultured for 72 h, then collected and washed with M9 buffer. *C. elegans* pellets were lysed by three freeze–thaw cycles in liquid nitrogen [14]. The TG content was determined using TG assay kits (Nanjing Jiancheng Bioengineering Institute, Nanjing, China) and normalized to the total protein concentration.

#### 2.5.4. Acute Oxidative and Heat Stress Assays

Synchronized L4-stage *C. elegans* were cultured in six groups for 72 h. For the oxidative stress assay, nematodes were transferred to food-free NGM plates containing 0.03% H_2_O_2_ (10 μL 30% H_2_O_2_ per 10 mL NGM) and incubated at 20 °C. For the heat stress assay, nematodes were transferred to food-free NGM plates and incubated at 37 °C. Each group contained three replicates per group (30 nematodes per group). Dead nematodes were recorded every hour until all had died. Death was determined as unresponsiveness to gentle prodding with a platinum wire. Mean lifespan was calculated as the average of the individual lifespans of all *C. elegans* in each group. Median and maximum lifespans were defined as the time when 50% and 0% of the *C. elegans* survived, respectively.

### 2.6. Network Pharmacology Analysis

The SwissTargetPrediction platform (http://www.swisstargetprediction.ch/, accessed on 7 January 2026) was applied to predict the potential targets of VPIIMH (Probability > 0). Disease-related targets were retrieved from the GeneCards database using “hyperlipidemia” (relevance score > 0.9) and “obesity” (relevance score > 7.0) as keywords. The intersection of the predicted VPIIMH targets with the disease targets yields the overlapping genes. The common targets were imported into the STRING database to obtain protein–protein interaction (PPI) data, and Cytoscape 3.9.1 was used to construct a PPI network and perform topological analysis. Core targets were selected based on betweenness centrality (BC) values (BC > 20). GO functional annotation and KEGG pathway enrichment analyses were performed on the selected targets.

### 2.7. Statistical Analysis

All experiments were performed in triplicate, and data were expressed as mean ± SD. Statistical analysis was performed using GraphPad Prism 9. Group differences were assessed by one-way ANOVA with the LDS post-hos test (*p* < 0.05). Mean, median, and maximum lifespans were calculated using SPSS 20.0 software, and survival curves were generated using GraphPad Prism 9.

## 3. Results and Discussion

### 3.1. Inhibitory Effect of VPIIMH on PL

#### 3.1.1. Inhibition of PL Activity by VPIIMH at Various Concentrations

**Figure 1:**
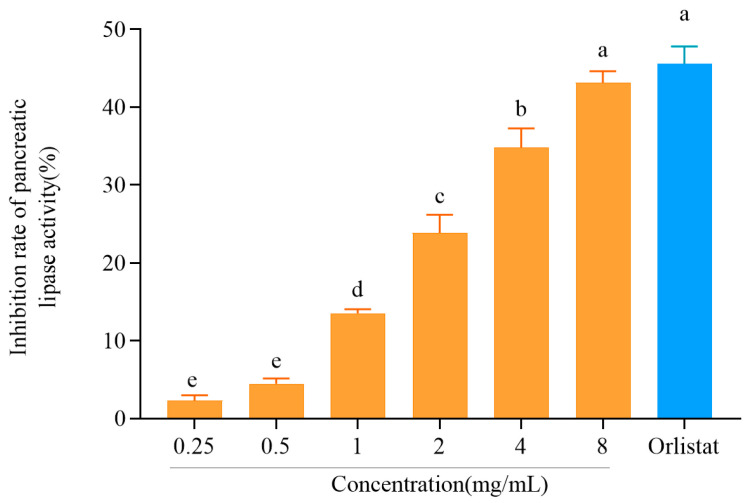
Pancreatic lipase inhibitory activity of VPIIMH at different concentrations. Different letters (a–e) above the bars indicate statistically signiffcant differences (*p* < 0.05) among the groups.

As shown in Figure 1, the inhibitory effect of VPIIMH on PL increased with concentration. The inhibition rates increased from 2.32 ± 0.66% to 43.17 ± 1.47% over a concentration range of 0.25–8.0 mg/mL. The clinically anti-obesity drug orlistat achieved 45.56 ± 2.26% inhibition at 8 μg/mL. Although the inhibitory activity of VPIIMH is lower than that of orlistat, these food-derived bioactive peptides offer more safety and suitability for consumption as a dietary supplement for long-term weight management, and this is consistent with its intended use. Notably, the activity of VPIIMH was comparable to that of FDTGSSFYNKPAG, the anti-obesity peptide isolated from heat-treated adzuki bean protein hydrolysate, which exhibited 36.28% inhibition at 4.0 mg/mL [17].

#### 3.1.2. Inhibition Kinetics of VPIIMH on PL

**Figure 2:**
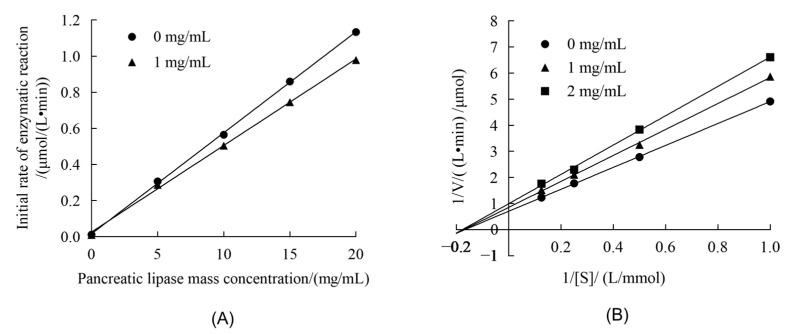
Inhibition kinetics of VPIIMH against pancreatic lipase. (**A**) Reversible inhibition assay and (**B**) Lineweaver–Burk plots.

The reaction velocity versus enzyme concentration plots for both groups passed through the origin, confirming that VPIIMH inhibits PL in a reversible manner (Figure 2A). In the Lineweaver–Burk plots, lines at different inhibitor concentrations (0, 1.0, and 2.0 mg/mL) intersected at a single point on the *x*-axis, with slopes increasing as the inhibitor concentration rose (Figure 2B). *V*_max_ decreased from 1.41 to 1.00 μmol/(L min). This characteristic demonstrated that VPIIMH inhibits PL in a non-competitive manner, with a *K*_m_ of 5.80 mmol/L. This mechanism aligns with that of the pentapeptide FLGPF previously derived from *Chlorella* [14]. Non-competitive implies that the inhibitory efficiency of VPIIMH is independent of substrate concentration, making it less likely to be overwhelmed by high dietary fat intake compared to competitive inhibitors.

### 3.2. Molecular Docking of VPIIMH with PL

**Figure 3:**
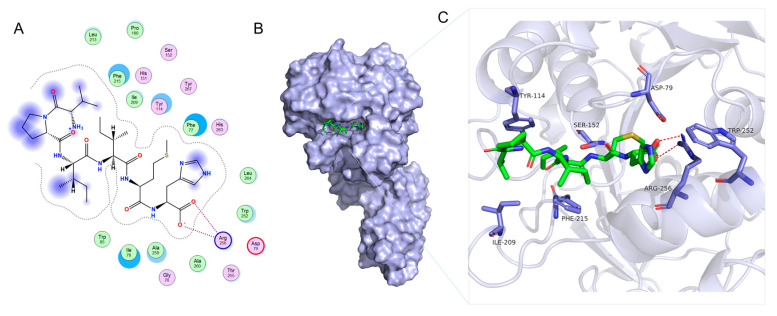
Molecular docking of VPIIMH with pancreatic lipase. (**A**) Two-dimensional interaction diagram; (**B**) surface representation of the binding pocket; (**C**) three-dimensional binding model. The peptide VPIIMH is colored in green. The surrounding residues in the binding pocket are colored in slate. The backbone of the receptor is depicted as light-blue cartoon. The ion contacts are depicted as red dashed lines.

Molecular docking analysis revealed that VPIIMH adopted a favorable spatial conformation with the PL crystal (Figure 3). The His6 residue of VPIIMH formed an ionic bond with Arg256 of PL via an oxygen–nitrogen interaction, while van der Waals forces further stabilized the complex. Notably, Arg256 locates outside the catalytic triad (Ser152, His263, and Asp176) of PL [18], indicating that VPIIMH binds to an allosteric site rather than competing with the substrate. This binding mode alters the enzyme conformation, reducing its activity without affecting substrate affinity, which is consistent with the inhibition kinetics observed in Figure 2B. In contrast, quinoa-derived peptides (LNEILAHE, VETELEKR, TVSAPSPK) bind to the PL active site via hydrogen bonds, electrostatic interactions, and hydrophobic interactions [19], while lotus seed-derived peptides (FLL and EFF) interact with the surrounding residues of the PL active site through hydrogen bonds and hydrophobic interactions [12]. These interactions typically result in competitive or mixed inhibition.

### 3.3. Evaluation of Lipid-Lowering Activity of VPIIMH in C. elegans

#### 3.3.1. Effects of VPIIMH on Fat Accumulation and TG Content

**Figure 4:**
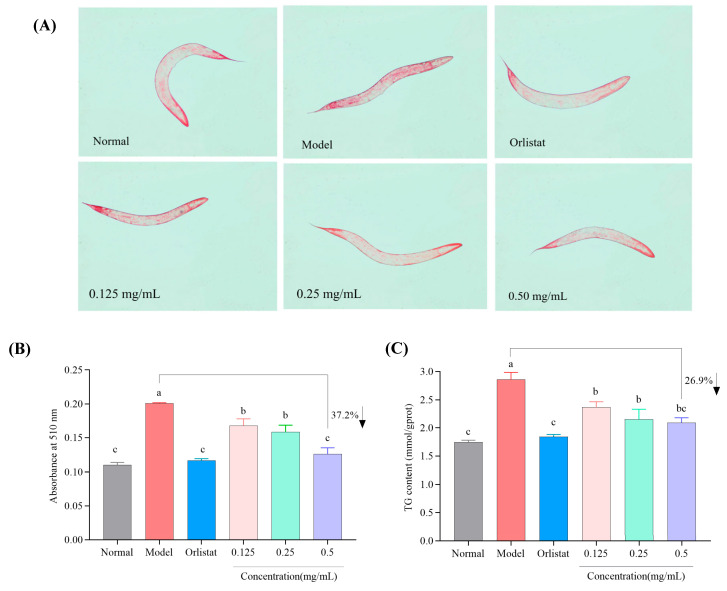
Effects of VPIIMH on fat accumulation and triglyceride levels in C. elegans. (**A**) Representative images of Oil Red O staining; (**B**) quantitative analysis of Oil Red O staining; (**C**) triglyceride content. Different letters (a–c) above the bars indicate statistically signiffcant differences (*p* < 0.05) among the groups.

Oil Red O staining was performed to evaluate the effect of VPIIMH on fat accumulation in *C. elegans*. This lipophilic dye penetrates the transparent body of the *C. elegans* and stains lipid droplets red, allowing for a quantitative assessment of fat content [15]. As shown in Figure 4A, *C. elegans* in the high-fat group exhibited markedly darker staining than the normal control, indicating a successful induction of fat accumulation by glucose. VPIIMH treatment progressively lightened staining with increasing concentrations, with the most pronounced effect at 0.5 mg/mL. The VPIIMH treatment staining intensity was intermediate between the normal and positive controls. Further quantitative analysis (Figure 4B) showed a significantly higher absorbance in the high-fat model group than in the normal control group (*p* < 0.05), confirming that 10 mM glucose effectively induced excessive fat accumulation. VPIIMH at 0.5 mg/mL reduced fat accumulation by 37.2% compared to the high-fat model group, showing an effect comparable to the positive control group (*p* > 0.05). These results demonstrate that VPIIMH exhibits potent lipid-lowering activity at a relatively low concentration (0.5 mg/mL). Its efficacy is comparable to previously reported peptides. Yu et al. [20] found that two peptides derived from *Sepia esculenta*, SeP2 (DVEDLEAGLAK) and SeP5 (EITSLAPSTM), decrease fat content in high-fat *C. elegans* by 20–25% at 4 mM.

As shown in Figure 4C, TG levels in the high-fat model group were significantly higher than those in the normal control (*p* < 0.05), confirming the successful induction of hyperlipidemia in *C. elegans* by glucose. The TG content of each VPIIMH treatment group at all doses significantly reduced the TG levels compared to the high-fat model group (*p* < 0.05), and with no significant difference among dose groups (*p* > 0.05). At 0.5 mg/mL, VPIIMH reduced TG by 26.9%, an effect comparable to the positive control (*p* > 0.05). This reduction is consistent with the fat accumulation profile observed by Oil Red O staining (Figure 4A,B).

#### 3.3.2. Enhancement of Antioxidant Stress Capacity by VPIIMH

**Figure 5:**
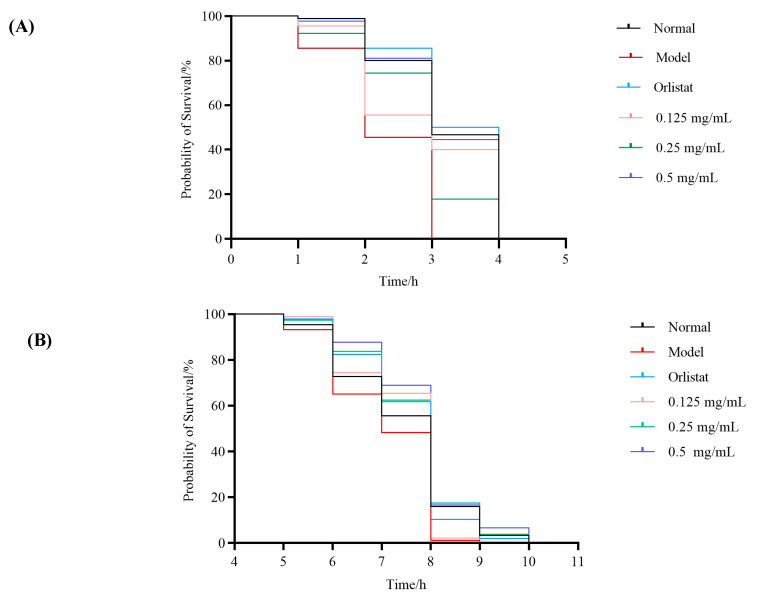
Survival curves of *C. elegans* under stress conditions. (**A**) Oxidative stress induced by H_2_O_2_ and (**B**) heat stress at 37 °C.

Oxidative stress is closely associated with obesity and its related diseases, such as diabetes, hypertension, dyslipidemia, and cardiovascular diseases [21]. Reducing oxidative stress is considered to contribute to alleviating obesity and its complications [20,22]. In this study, the ability of VPIIMH to enhance oxidative stress resistance was evaluated in high-fat *C. elegans* using an H_2_O_2_-induced acute oxidative stress model. As shown in Table 2 and Figure 5A, VPIIMH treatment significantly enhanced the survival of high-fat *C. elegans* under oxidative stress in a dose-dependent manner. Compared to the high-fat model group (2.31 ± 0.15 h), treatment with VPIIMH at 0.5 mg/mL extended the mean lifespan to 3.24 ± 0.23 h, an increase of 40.3% (*p* < 0.05), which was comparable to that of the positive control (*p* > 0.05). The median lifespan also increased progressively with VPIIMH concentration, and the survival curves shifted rightward, further confirming the protective effect against oxidative damage. These findings demonstrate that VPIIMH possesses the ability to enhance the antioxidant capacity of *C. elegans*, which may contribute to its lipid-lowering effects by mitigating the oxidative stress associated with fat accumulation.

#### 3.3.3. Enhancement of Heat Stress Resistance by VPIIMH

Heat stress triggers a cascade of adaptive and potentially damaging responses in organisms, and when stress exceeds the adaptive capacity, it can lead to metabolic dysregulation, tissue damage, and impairment of physiological function [23,24]. Given the impact of heat stress and lipid metabolism, the effect of VPIIMH on heat stress resistance was evaluated in high-fat *C. elegans* exposed to 37 °C. As shown in Table 3 and Figure 5B, VPIIMH treatment dose-dependently improved the survival of high-fat *C. elegans* under heat. Compared to the high-fat model group, VPIIMH at 0.5 mg/mL extended the mean lifespan by 17.5% (*p* < 0.05), while the increases in median lifespan and maximum lifespan were not significant (*p* > 0.05). The survival curves shifted progressively rightward with increasing VPIIMH concentrations, with the strongest effect observed at 0.5 mg/mL. These findings indicate that VPIIMH enhances heat stress resistance in high-fat *C. elegans*, contributing to its protective effects against lipid accumulation.

### 3.4. Network Pharmacology Analysis of Potential Lipid-Lowering Mechanisms of VPIIMH

The in vitro and in vivo results described above demonstrated that VPIIMH possesses lipid-lowering and anti-obesity properties. Specifically, VPIIMH not only directly inhibits LP activity but also significantly reduces the TG levels and fat accumulation in high-glucose diet-induced *C. elegans*, while enhancing their resistance to oxidative and heat stress. However, its potential molecular targets and mechanisms of action in mammals, and ultimately in humans, remain unclear. To address this gap, we utilized network pharmacology to systematically predict the potential targets and regulation pathways modulated by VPIIMH, providing a theoretical basis for elucidating its lipid-lowering mechanism.

#### 3.4.1. Prediction of VPIIMH Potential Targets Related to Lipid-Lowering

**Figure 6:**
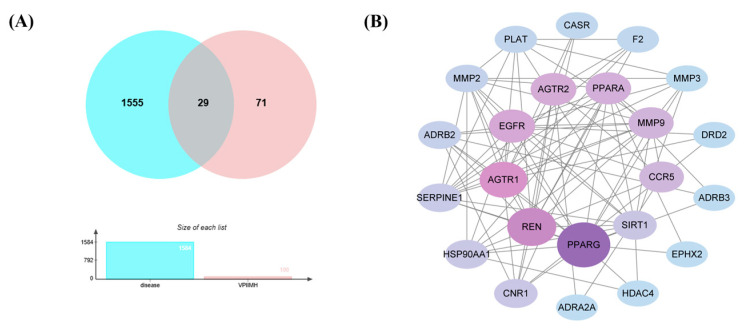
Network pharmacology analysis of VPIIMH targets. (**A**) Venn diagram of VPIIMH and disease-related targets. Blue: hyperlipidemia/obesity‑related targets; pink: VPIIMH targets. (**B**) PPI network of potential targets. Note size is proportional to betweenness centrality (BC).

By intersecting the predicted VPIIMH targets with the disease-related targets, 29 overlapping genes were obtained (Figure 6A). To explore the interactions among these candidates, the 29 overlapping genes were imported into the STRING database to construct a PPI network. After removing isolated nodes with no interactions, a PPI network consisting of 23 nodes and 90 edges was generated (Figure 6B). The network exhibited a high degree of connectivity (with an average node degree of 7.83), suggesting that VPIIMH may exert its lipid-lowering effects through multi-target synergy and pathway integration.

These 23 targets were ranked based on betweenness centrality (BC), with the values listed in Table 4. The top eight targets with the highest BC values were PPARG (94.61), REN (60.10), AGTR1 (49.05), EGFR (35.56), AGTR2 (32.86), PPARA (30.10), MMP9 (27.04), and CCR5 (24.35). Notably, a significant drop in BC value was observed between the eighth (CCR5, 24.35) and the ninth (SIRT1, 15.07) ranked target, representing a 38.1% decrease. This indicates that the top eight targets constitute the “core hub” of the network and were selected as core nodes (BC > 20) for subsequent analysis. Additionally, SIRT1 plays a crucial role in energy metabolism and lifespan regulation, and it has a strong correlation with the observed phenotypes of enhanced oxidative stress resistance (+40.3%) and heat stress tolerance (+17.5%) in VPIIMH-treated *C. elegans*, despite its lower BC value.

**Figure 7:**
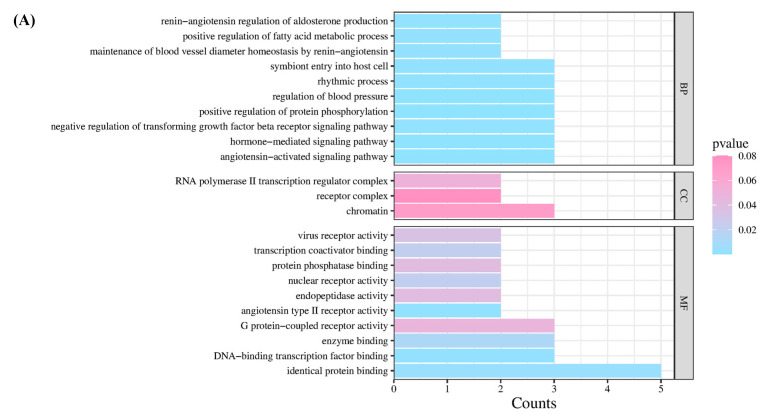

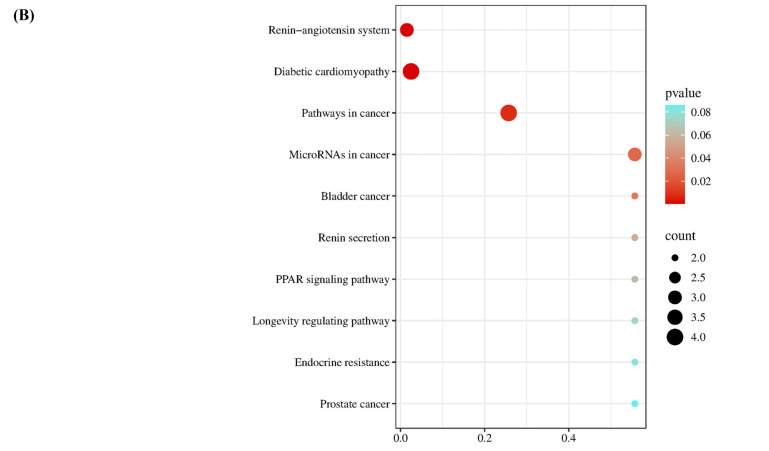
Functional enrichment analysis of key targets. (**A**) GO enrichment analysis. BP: biological process; CC: cellular component; MF: molecular function. (**B**) KEGG pathway enrichment analysis.

To further elucidate the biological functions of these nine core targets, GO enrichment and KEGG pathway analyses were performed. GO analysis revealed that the targets were significantly enriched in biological processes involved in angiotensin-activated signaling pathways, hormone-mediated signaling pathways, positive regulation of fatty acid metabolic processes, and involved in molecular functions, including angiotensin type II receptor activity and DNA-binding transcription factor binding (Figure 7A). KEGG pathway enrichment analysis showed that the nine core targets were significantly enriched in five signaling pathways (*p* < 0.05), including the renin-angiotensin system (RAS), diabetic cardiomyopathy, pathways in cancer, microRNAs in cancer, and bladder cancer (Figure 7B). These results suggest that VPIIMH may exert its lipid-lowering and anti-obesity effects through a multi-pathway regulatory network.

#### 3.4.2. Potential VPIIMH-Mediated Lipid Regulation Mechanism

To further elucidate the molecular basis of the multi-target lipid-lowering effect of VPIIMH, the nine core targets were categorized into three independent yet synergistic regulatory modules based on their biological functions.

Lipid metabolism homeostasis regulation mediated by the SIRT1-PPAR axis (the core regulatory module): PPARG (BC = 94.61) and PPARA (BC = 30.10) are key nodes in the PPAR signaling pathway. PPARG, as the master transcriptional regulator of adipogenesis, controls adipocyte differentiation and lipid synthesis [25,26]. Conversely, PPARA enhances hepatic β-oxidation, reducing hepatic lipid accumulation [27]. Together, they establish a balance between “lipogenesis” and “lipolysis”, which is central to maintaining lipid homeostasis. SIRT1 serves as a key node in the regulation of energy homeostasis, acting through an “inhibit synthesis, promote catabolism” regulatory pattern. It regulates the transcriptional activity of the PPAR family via deacetylation [28], simultaneously inhibiting PPARA-mediated adipogenesis and promoting PPARA-driven fatty acid oxidation [29]. This regulatory pattern aligns closely with our phenotypic observations, where VPIIMH reduced TG levels by 26.9% and fat accumulation by 37.2% in *C. elegans*. SIRT1 also activates the FOXO transcription factor, upregulating antioxidant enzymes (SOD, CAT) and inhibiting the NF-κB inflammatory pathway [29,30], thereby providing a molecular basis for explaining its anti-stress effects.

Metabolic regulation of the RAS (co-regulation module): REN, AGTR1, and AGTR2 are key nodes of the renin-angiotensin system (RAS), and their functional imbalance is a major contributor to lipid metabolic disorders [31,32]. As the rate-limiting enzyme, REN drives RAS overactivation by catalyzing the production of angiotensin II (Ang II). Excessive Ang II binding to AT1R (encoded by AGTR1) promotes adipogenesis and inhibits fatty acid oxidation, leading to triglyceride accumulation and obesity-related pathologies [33,34]. In contrast, Ang II binding to AT2R (encoded by AGTR2) counteracts these effects, helping to maintain metabolic homeostasis [34]. By modulating REN activity and the AGTR1/AGTR2 balance, VPIIMH may suppress RAS overactivation and restore lipid metabolic homeostasis at the systemic level.

Inflammatory targets and adipose tissue remodeling (Microenvironment regulation module): Obesity is accompanied by sustained low-grade inflammation within adipose tissue. EGFR, MMP9, and CCR5 play pivotal roles in shaping this inflammation. CCR5 mediates the chemotaxis and infiltration of immune cells into adipose tissue [35], and MMP9 degrades extracellular matrix components; its excessive activation leads to adipose tissue fibrosis and remodeling, facilitating inflammatory cell infiltration [36]. EGFR is expressed in adipocyte tissue macrophages, and its activation promotes macrophage proliferation, monocyte infiltration into adipose tissue, and proinflammatory cytokine release, leading to the exacerbation of local inflammation and insulin resistance [37,38]. CCR5, MMP9, and EGFR contribute to immune cell infiltration, extracellular matrix remodeling, and adipocyte dysfunction, respectively, perpetuating the vicious cycle of “lipid accumulation—inflammation—metabolic disorder”. VPIIMH may ameliorate the adipose tissue microenvironment by inhibiting these targets, creating favorable conditions for core metabolic regulation.

Collectively, VPIIMH exerts its lipid-lowering effect through a multi-level, multi-targeted synergistic network. First, centered on the SIRT1-PPAR axis, it directly drives the “inhibit synthesis, promoting catabolism” metabolic reprogramming and activates the SIRT1-mediated stress defense. This directly accounts for the observed reduction in TG levels (−26.9%) and fat accumulation (−37.2%), as well as the enhanced stress resistance (oxidative stress lifespan +40.3%, heat stress lifespan +17.5%). Second, by regulating REN/AGTR1/AGTR2 balance, it synergistically modulates lipid metabolism at the systemic level. Finally, by inhibiting CCR5, MMP9, and EGFR, it improves adipose tissue inflammatory microenvironment, thereby disrupting the self-perpetuating cycle of metabolic dysregulation. This “core-driven, system-coordinated, microenvironment-supportive” tripartite regulatory model provides new molecular insights into the potential of VPIIMH as a functional food ingredient for combating obesity and related metabolic disorders.

However, the exclusive use of the *C. elegans* model represents a limitation; further validation in mammalian models is essential to assess efficacy and safety for human use. Given that VPIIMH is a food-derived bioactive peptide with reversible, lipid-lowering and anti-stress effects, it represents an attractive candidate for incorporation into functional food products aimed at weight management and metabolic health. Future work should also evaluate its stability in food matrices, oral bioavailability, and potential synergistic effects with other nutraceuticals. Such studies will further support the translation of this *Chlorella*-derived peptide into commercial functional food products.

## 4. Conclusions

This study demonstrates that *Chlorella*-derived hexapeptide VPIIMH possesses significant lipid-lowering and anti-obesity properties through a multi-target mechanism. In vitro, VPIIMH acts as a reversible non-competitive inhibitor of PL, indicating its potential to reduce dietary fat absorption. In a high-fat *C. elegans* model, VPIIMH not only reduced fat accumulation and TG levels, but also enhanced resistance to oxidative and heat stress, revealing a dual function of lipid-lowering and anti-stress. Network pharmacology analysis further predicted that VPIIMH exerts multi-target regulatory effects through the SIRT1-PPAR axis, the RAS, and inflammatory-related targets (CCR5, MMP9, EGFR). This “core-driven—system-coordinated—microenvironment” tripartite regulatory model provides a mechanistic framework for understanding the multi-target action of VPIIMH. Nevertheless, the observed dual effects suggest that VPIIMH may act through both direct enzyme inhibition and activation of endogenous defense pathways. These findings establish VPIIMH as a promising candidate for functional food applications targeting obesity and related metabolic disorders.

## Figures and Tables

**Table 1:** Experiment groups and VPIIMH concentration used in the *C. elegans* assay.

Group	Medium	Drug Feeding
Normal	NGM	OP50 without drugs
Model	NGM + 10 mmol/Lglucose	OP50 without drugs
Orlistat	NGM + 10 mmol/Lglucose	OP50 with Orlistat (6 μg/mL)
0.125 mg/mL	NGM + 10 mmol/Lglucose	OP50 with VPIIMH (0.125 mg/mL)
0.25 mg/mL	NGM + 10 mmol/Lglucose	OP50 with VPIIMH (0.25 mg/mL)
0.50 mg/mL	NGM + 10 mmol/Lglucose	OP50 with VPIIMH (0.5 mg/mL)

**Table 2:** Effect of VPIIMH on the survival of high-fat *C. elegans* under oxidation stress.

Group	Mean Lifespan/h	Median Lifespan/h	Maximun Lifespan/h
Normal	3.26 ± 0.13 ^a^	3.33 ± 0.58 ^a^	4
Model	2.31 ± 0.15 ^c^	2.50 ± 0.50 ^a^	3
Orlistat	3.32 ± 0.15 ^a^	3.33 ± 0.58 ^a^	4
0.125 mg/mL	2.50 ± 0.09 ^bc^	2.83 ± 0.76 ^a^	4
0.25 mg/mL	2.84 ± 0.02 ^b^	3.00 ± 0.00 ^a^	4
0.50 mg/mL	3.24 ± 0.23 ^a^	3.33 ± 0.58 ^a^	4

Different letters (a–c) represent a statistical difference between groups (*p* < 0.05).

**Table 3:** Effect of VPIIMH on the survival of high-fat *C. elegans* under heat stress.

Group	Mean Lifespan/h	Median Lifespan/h	Maximun Lifespan/h
Normal	7.40 ± 0.85 ^ab^	7.33 ± 1.15 ^a^	9.33 ± 1.15 ^a^
Model	7.01 ± 0.37 ^b^	6.67 ± 0.76 ^a^	8.33 ± 0.58 ^a^
Orlistat	7.52 ± 0.41 ^ab^	8.00 ± 0.00 ^a^	9.00 ± 1.00 ^a^
0.125 mg/mL	7.41 ± 0.22 ^ab^	8.00 ± 0.00 ^a^	8.33 ± 0.58 ^a^
0.25 mg/mL	7.69 ± 0.20 ^ab^	8.00 ± 0.00 ^a^	9.33 ± 1.15 ^a^
0.50 mg/mL	8.24 ± 0.24 ^a^	8.00 ± 0.00 ^a^	10.00 ± 0.00 ^a^

Different letters (a,b) represent a statistical difference between groups (*p* < 0.05).

**Table 4:** Topological parameters of the 23 candidate targets in the PPI network.

Gene Symbol	Gene Name	Classification	Probability	BC Values
PPARG	Peroxisome proliferator-activated receptor gamma	Nuclear receptor	0.060	94.61
REN	Renin	Protease	0.060	60.10
AGTR1	Type-1 angiotensin II receptor	Family A G protein-coupled receptor	0.060	49.05
EGFR	Epidermal growth factor receptor erbB1	Kinase	0.060	35.56
AGTR2	Angiotensin II receptor	Family A G protein-coupled receptor	0.060	32.86
PPARA	Peroxisome proliferator-activated receptor alpha	Nuclear receptor	0.060	30.10
MMP9	Matrix metalloproteinase 9	Protease	0.060	27.04
CCR5	C-C chemokine receptor type 5	Family A G protein-coupled receptor	0.060	24.35
SIRT1	NAD-dependent deacetylase sirtuin 1	Eraser	0.116	15.07
CNR1	Cannabinoid receptor 1 (by homology)	Family A G protein-coupled receptor	0.060	14.51
HSP90AA1	Heat shock protein HSP 90-alpha	Other cytosolic protein	0.060	13.88
SERPINE1	Plasminogen activator inhibitor-1	Secreted protein	0.060	12.10
ADRB2	Adrenergic receptor beta	Family A G protein-coupled receptor	0.060	8.98
MMP2	Matrix metalloproteinase 2	Protease	0.060	7.07
PLAT	Tissue-type plasminogen activator	Protease	0.060	4.93
CASR	Calcium-sensing receptor	Family C G protein-coupled receptor	0.060	3.33
F2	Thrombin	Protease	0.060	2.47
MMP3	Matrix metalloproteinase 3	Protease	0.060	0.92
DRD2	Dopamine D2 receptor (by homology)	Family A G protein-coupled receptor	0.060	0.40
ADRB3	Beta-3 adrenergic receptor	Family A G protein-coupled receptor	0.060	0.33
ADRA2A	Alpha-2a adrenergic receptor	Family A G protein-coupled receptor	0.060	0.00
EPHX2	Epoxide hydratase	Protease	0.060	0.00
HDAC4	Histone deacetylase 4	Eraser	0.060	0.00

## Data Availability

The original contributions presented in this study are included in the article. Further inquiries can be directed to the corresponding author.

## References

[1] Zhang X., Zhang B., Zhang C., Sun G., Sun X. (2020). Effect of *Panax notoginseng* Saponins and Major Anti-Obesity Components on Weight Loss. Front. Pharmacol..

[2] Zhou C., Yin X. (2022). Wogonin Ameliorated Obesity-Induced Lipid Metabolism Disorders and Cardiac Injury via Suppressing Pyroptosis and Deactivating IL-17 Signaling Pathway. Am. J. Chin. Med..

[3] Bjornsson E.S. (2017). Hepatotoxicity of statins and other lipid-lowering agents. Liver Int..

[4] D’Souza K., Mercer A., Mawhinney H., Pulinilkunnil T., Udenigwe C.C., Kienesberger P.C. (2020). Whey Peptides Stimulate Differentiation and Lipid Metabolism in Adipocytes and Ameliorate Lipotoxicity-Induced Insulin Resistance in Muscle Cells. Nutrients.

[5] Li T., Han K., Feng G., Guo J., Wan Z., Yang X. (2024). Condensation of Soy Protein Peptides Contributes to Sequester Bile Acids and Mitigate LPS-Induced Inflammation. J. Agric. Food Chem..

[6] Affane F., Louala S., El Imane Harrat N., Bensalah F., Chekkal H., Allaoui A., Lamri-Senhadji M. (2018). Hypolipidemic, antioxidant and antiatherogenic property of sardine by-products proteins in high-fat diet induced obese rats. Life Sci..

[7] Chen H., Cheng S., Fan F., Tu M., Xu Z., Du M. (2019). Identification and molecular mechanism of antithrombotic peptides from oyster proteins released in simulated gastro-intestinal digestion. Food Funct..

[8] Sergi D., Melloni M., Passaro A., Neri L.M. (2024). Influence of Type 2 Diabetes and Adipose Tissue Dysfunction on Breast Cancer and Potential Benefits from Nutraceuticals Inducible in Microalgae. Nutrients.

[9] Zhang R., Chen J., Mao X., Qi P., Zhang X. (2019). Separation and Lipid Inhibition Effects of a Novel Decapeptide from *Chlorella pyenoidose*. Molecules.

[10] Wang D., Lin L., Liang P., Liu W., Ma W., Wang Y., Chen J. (2025). Exploring the Novel Pancreatic Lipase-Inhibitory Peptides in *Chlorella pyrenoidosa*: Preparation, Purification, Identification, and Molecular Docking. Foods.

[11] Rajan L., Palaniswamy D., Mohankumar S.K. (2020). Targeting obesity with plant-derived pancreatic lipase inhibitors: A comprehensive review. Pharmacol. Res..

[12] Chen H., Lang Z., Chen J., Gao T., Zheng B., Zeng S. (2025). Novel pancreatic lipase inhibitory peptides derived from lotus seed protein: Isolation, identification, and the interaction mechanism. Food Funct..

[13] Kim H., Jeon Y.E., Kim S.M., Jung J.I., Ko D., Kim E.J. (2023). Agaricus bisporus Extract Exerts an Anti-Obesity Effect in High-Fat Diet-Induced Obese C57BL/6N Mice by Inhibiting Pancreatic Lipase-Mediated Fat Absorption. Nutrients.

[14] Luan L., Wenjun L., Junyuan H., Aijing J., Dengmi W., Bin1 L., Chao Z. (2023). Isolation, Purification, Identification and Hypolipidemic Activity of Lipase Inhibitory Peptide from *Chlorella pyrenoidosa*. Food Sci..

[15] Liu L., Kong Q., Xiang Z., Kuang X., Wang H., Zhou L., Feng S., Chen T., Ding C. (2023). Integrated Analysis of Transcriptome and Metabolome Provides Insight into Camellia oleifera Oil Alleviating Fat Accumulation in High-Fat *Caenorhabditis elegans*. Int. J. Mol. Sci..

[16] Li A.P., Li D., Tan X., Xu R., Mao L.X., Kang J.J., Li S.H., Liu Y. (2025). Crocin extends lifespan by mitigating oxidative stress and regulating lipid metabolism through the DAF-16/FOXO pathway. Food Funct..

[17] Zhao Q., Fan Y., Zhao L., Zhu Y., Jiang Y., Gu J., Xue Y., Hao Z., Shen Q. (2024). Identification and molecular binding mechanism of novel pancreatic lipase and cholesterol esterase inhibitory peptides from heat-treated adzuki bean protein hydrolysates. Food Chem..

[18] Bhaskaran A., Aitken H.M., Xiao Z., Blyth M., Nothling M.D., Kamdar S., O’Mara M.L., Connal L.A. (2021). Enzyme inspired polymer functionalized with an artificial catalytic triad. Polymer.

[19] Fu Z., Wang J., Wu C., Qin J., Lee B.H., Li M., Sun Q., Tang W. (2025). Targeted screening of pancreatic lipase inhibitory peptides from quinoa protein hydrolysates by ligand fishing. Food Chem..

[20] Yu X., Su Q., Shen T., Chen Q., Wang Y., Jia W. (2020). Antioxidant Peptides from *Sepia esculenta* Hydrolyzate Attenuate Oxidative Stress and Fat Accumulation in *Caenorhabditis elegans*. Mar. Drugs.

[21] Olivares-Vicente M., Herranz-López M. (2025). The Interplay Between Oxidative Stress and Lipid Composition in Obesity-Induced Inflammation: Antioxidants as Therapeutic Agents in Metabolic Diseases. Int. J. Mol. Sci..

[22] Cao K., Wang K., Yang M., Liu X., Lv W., Liu J. (2020). Punicalagin improves hepatic lipid metabolism via modulation of oxidative stress and mitochondrial biogenesis in hyperlipidemic mice. Food Funct..

[23] Zhao T., Ma A., Yang S., Huang Z. (2021). Integrated metabolome and transcriptome analyses revealing the effects of thermal stress on lipid metabolism in juvenile turbot *Scophthalmus maximus*. J. Therm. Biol..

[24] Yasoob T.B., Khalid A.R., Zhang Z., Zhu X., Hang S. (2022). Liver transcriptome of rabbits supplemented with oral *Moringa oleifera* leaf powder under heat stress is associated with modulation of lipid metabolism and up-regulation of genes for thermo-tolerance, antioxidation, and immunity. Nutr. Res..

[25] Li J., Guo M., Yuan C., Li T., Zhang J., Ren L. (2024). Ameliorative potential of ellagic acid via PPARγ against hyperlipidemia: Insights from mice and zebrafish. Food Biosci..

[26] Li X., Tan K.S.W., Wang L. (2025). Black rice bran improved lipid metabolism in hyperlipidemia mice via steroid hormone biosynthesis and PPAR signaling pathway. J. Pharm. Biomed. Anal..

[27] Fatriani R., Pratiwi F.A.K., Annisa A., Septaningsih D.A., Aziz S.A., Miladiyah I., Kusumastuti S.A., Nasution M.A.F., Ramadhan D., Kusuma W.A. (2024). Unveiling the anti-obesity potential of Kemuning (*Murraya paniculata*): A network pharmacology approach. PLoS ONE.

[28] Shen S., Shen M., Kuang L., Yang K., Wu S., Liu X., Wang Y., Wang Y. (2024). SIRT1/SREBPs-mediated regulation of lipid metabolism. Pharmacol. Res..

[29] Zhang W., Sun Y., Liu W., Dong J., Chen J. (2019). SIRT1 mediates the role of RNA-binding protein QKI 5 in the synthesis of triglycerides in non-alcoholic fatty liver disease mice via the PPARα/FoxO1 signaling pathway. Int. J. Mol. Med..

[30] Wu Q.J., Zhang T.N., Chen H.H., Yu X.F., Lv J.L., Liu Y.Y., Liu Y.S., Zheng G., Zhao J.Q., Wei Y.F. (2022). The sirtuin family in health and disease. Signal Transduct. Target. Ther..

[31] Tiao M.M., Lin Y.J., Yu H.R., Sheen J.M., Lin I.C., Lai Y.J., Tain Y.L., Huang L.T., Tsai C.C. (2018). Resveratrol ameliorates maternal and post-weaning high-fat diet-induced nonalcoholic fatty liver disease via renin-angiotensin system. Lipids Health Dis..

[32] Yang M., Ma X., Xuan X., Deng H., Chen Q., Yuan L. (2020). Liraglutide Attenuates Non-Alcoholic Fatty Liver Disease in Mice by Regulating the Local Renin-Angiotensin System. Front. Pharmacol..

[33] Simões e Silva A.C., Miranda A.S., Rocha N.P., Teixeira A.L. (2017). Renin angiotensin system in liver diseases: Friend or foe. World J. Gastroenterol..

[34] White M.C., Fleeman R., Arnold A.C. (2019). Sex differences in the metabolic effects of the renin-angiotensin system. Biol. Sex Differ..

[35] Li R., Wang R., Li L., Wang C., Liao T., Zhang B., Yue R. (2025). Shared Therapeutic Targets of Obesity and Type 2 Diabetes Mellitus and the Intervention Mechanisms of Chinese Herbal Components. Sichuan Da Xue Xue Bao Yi Xue Ban..

[36] Taheri A., Mobaser S.E., Golpour P., Nourbakhsh M., Tavakoli-Yaraki M., Yarahmadi S., Nourbakhsh M. (2023). Hesperetin attenuates the expression of markers of adipose tissue fibrosis in pre-adipocytes. BMC Complement. Med. Ther..

[37] Cao S., Pan Y., Tang J., Terker A.S., Arroyo Ornelas J.P., Jin G.N., Wang Y., Niu A., Fan X., Wang S. (2022). EGFR-mediated activation of adipose tissue macrophages promotes obesity and insulin resistance. Nat. Commun..

[38] Zheng L.-L., Wang L., Guo C., He Y.-F., Xie J.-J., Ma Y.-H. (2024). Effect of Cigu Xiaozhi decoction on EGFR/PI3K/AKT signaling pathway in NASH’s “inflammatory cancer” transformation based on network pharmacology and animal experiments. Chin. Pharmacol. Bull..

